# Assessing the Reliability of the OMERACT Juvenile Idiopathic Arthritis Magnetic Resonance Scoring System for Temporomandibular Joints (JAMRIS-TMJ)

**DOI:** 10.3390/jcm10184047

**Published:** 2021-09-07

**Authors:** Mirkamal Tolend, Andrea S. Doria, Arthur B. Meyers, Tore A. Larheim, Shelly Abramowicz, Julien Aguet, Simone Appenzeller, Linda Z. Arvidsson, Lauren W. Averill, Brian M. Feldman, Saurabh Guleria, Emilio J. Inarejos Clemente, Jacob L. Jaremko, Thitiporn Junhasavasdikul, Thekla von Kalle, Eva Kirkhus, Bernd Koos, Elka Miller, Rahim Moineddin, Jyoti Panwar, Zachary S. Peacock, Cory M. Resnick, Marion A. van Rossum, Jennifer Stimec, George Tomlinson, Nikolay Tzaribachev, Christian J. Kellenberger

**Affiliations:** 1 Institute of Medical Science, University of Toronto, Toronto, ON M5S 1A8, Canada; m.tolend@mail.utoronto.ca (M.T.); brian.feldman@sickkids.ca (B.M.F.); 2 Department of Diagnostic Imaging, The Hospital for Sick Children, Toronto, ON M5G 1X8, Canada; Julien.Aguet@sickkids.ca (J.A.); jennifer.stimec@sickkids.ca (J.S.); 3 Department of Radiology, Cincinnati Children’s Hospital Medical Center, Cincinnati, OH 45229, USA; arthur.meyers@cchmc.org; 4 Department of Maxillofacial Radiology, Institute of Clinical Dentistry, University of Oslo, 0317 Oslo, Norway; t.a.larheim@odont.uio.no (T.A.L.); l.z.arvidsson@odont.uio.no (L.Z.A.); 5 Division of Oral and Maxillofacial Surgery, Departments of Surgery and Pediatrics, Emory University School of Medicine, Atlanta, GA 30322, USA; sabram5@emory.edu; 6 Oral and Maxillofacial Surgery, Children’s Healthcare of Atlanta, Atlanta, GA 30322, USA; 7 Department of Orthopedics, Rheumatology and Traumatology, School of Medical Science, University of Campinas, Campinas 13083-970, Brazil; appenzellersimone@yahoo.com; 8 Department of Medical Imaging, Nemours Children’s Health System, Alfred I. duPont Hospital for Children, Wilmington, DE 19803, USA; lauren.averill@nemours.org; 9 Division of Rheumatology, The Hospital for Sick Children, Toronto, ON M5G 1X8, Canada; 10 Austin Radiological Association, Austin, TX 78731, USA; saurabhguleria@gmail.com; 11 Department of Diagnostic Imaging, Hospital Sant Joan de Deu, 08950 Barcelona, Spain; einarejos@sjdhospitalbarcelona.org; 12 Department of Radiology & Diagnostic Imaging, University of Alberta, Edmonton, AB T6G 2B7, Canada; jjaremko@ualberta.ca; 13 Faculty of Medicine Ramathibodi Hospital, Mahidol University, Bangkok 10400, Thailand; maprangpo@hotmail.com; 14 Department of Pediatric Radiology, Radiologisches Institut, Olgahospital Klinikum Stuttgart, 70174 Stuttgart, Germany; t.vonkalle@klinikum-stuttgart.de; 15 Department of Radiology, Oslo University Hospital, 0424 Oslo, Norway; eva.kirkhus@ous-hf.no; 16 Department of Orthodontics, University Hospital Tübingen, 72076 Tübingen, Germany; Bernd.Koos@med.uni-tuebingen.de; 17 Department of Medical Imaging, Children’s Hospital of Eastern Ontario, University of Ottawa, Ottawa, ON K1H 8L1, Canada; EMiller@cheo.on.ca; 18 Department of Family & Community Medicine, University of Toronto, Toronto, ON M5G 1V7, Canada; rahim.moineddin@utoronto.ca; 19 Department of Radiology, Christian Medical College and Hospital, Vellore 632004, Tamil Nadu, India; drjyoticmch@gmail.com; 20 Department of Oral and Maxillofacial Surgery, Massachusetts General Hospital, Boston, MA 02114, USA; zpeacock@partners.org; 21 Department of Plastic and Oral Surgery, Boston Children’s Hospital, Boston, MA 02115, USA; Cory.Resnick@childrens.harvard.edu; 22 Department of Pediatrics, Emma Children’s Hospital, Amsterdam University Medical Center, 1105 AZ Amsterdam, The Netherlands; m.a.vanrossum@amsterdamumc.nl; 23 Amsterdam Rheumatology and Immunology Center, Reade, 1007 MB Amsterdam, The Netherlands; 24 Department of Medicine, University Health Network, Toronto, ON M5G 2C4, Canada; george.tomlinson@utoronto.ca; 25 Pediatric Rheumatology Research Institute, 24576 Bad Bramstedt, Germany; tzaribachev@pri-research.com; 26 Department of Diagnostic Imaging, University Children’s Hospital Zürich, 8032 Zürich, Switzerland; Christian.Kellenberger@kispi.uzh.ch

**Keywords:** juvenile idiopathic arthritis, magnetic resonance imaging, temporomandibular joints, outcome measure, reliability, generalizability theory

## Abstract

Contrast-enhanced magnetic resonance imaging (MRI) remains the most comprehensive modality to assess juvenile idiopathic arthritis (JIA)-related inflammation and osteochondral damage in the temporomandibular joints (TMJ). This study tested the reliability of a new JIA MRI scoring system for TMJ (JAMRIS-TMJ) and the impact of variations in calibration and reader specialty. Thirty-one MRI exams of bilateral TMJs were scored independently using the JAMRIS-TMJ by 20 readers consisting of radiologists and non-radiologist clinicians in three reading groups, with or without a calibrating atlas and/or tutorial. The inter-reader reliability in the multidisciplinary cohort assessed by the generalizability coefficient was 0.61–0.67 for the inflammatory and 0.66–0.74 for the damage domain. The atlas and tutorial did not improve agreement within radiologists, but improved the agreement between radiologist and non-radiologist groups. Agreements between different calibration levels were 0.02 to 0.08 lower by the generalizability coefficient compared to agreement within calibration levels; agreement between specialty groups was 0.04 to 0.10 lower than within specialty groups. Averaging two radiologists raised the reliability above 0.8 for both domains. Therefore, the reliability of JAMRIS-TMJ was moderate-to-good depending on the presence of specialty and calibration differences. The atlas and tutorial are necessary to improve reliability when the reader cohort consists of multiple specialties.

## 1. Introduction

There is an increasing need to standardize the imaging assessment of temporomandibular joints (TMJ) in children with juvenile idiopathic arthritis (JIA). The involvement of the TMJ in JIA is frequent yet often difficult to detect clinically at early stages. The reported frequency of TMJ involvement in large series varies between 40 and 70% [1,2,3,4]. These changes often develop without clinical findings, yet may lead to irreversible facial changes and functional impairments in severe cases [5,6,7,8]. The effective use of TMJ imaging is therefore important for enabling earlier disease detection and the start of medical, orthodontic, orthopedic, and physiotherapeutic management to prevent or minimize severe functional outcomes. Contrast enhanced magnetic resonance imaging (MRI) is currently the most informative imaging modality, as it allows visualization of both the active inflammatory disease as well as the extent of structural damage in the TMJ. Other imaging modalities cannot comprehensively assess both domains of disease burden [1,3,9,10,11,12,13,14,15]. However, there remains great variability in the acquisition and interpretation of TMJ MRI.

An international, multidisciplinary expert group was formed within the Outcome Measures in Rheumatology (OMERACT) network to develop MRI scoring systems for JIA (JAMRI working group), with a dedicated subgroup for developing the TMJ-specific scoring system (JAMRIS-TMJ). A consensus scoring system was drafted based on the testing of existing TMJ MRI scoring systems and subsequent formal consensus techniques, including Delphi surveys, nominal group technique, and consensus voting [16]. Relative importance weights of the items and grades were determined through a discrete choice experiment method and were shown to possess face validity and construct validity in an image-based vignette ranking exercise [17]. The present study was undertaken as the next step in testing the required clinimetric properties of the weighted JAMRIS-TMJ, specifically its reliability, in line with the instrument appraisal framework of OMERACT [18].

In this study, we examined the reliability of the semiquantitative JAMRIS-TMJ scoring system with a large multicenter, multidisciplinary group of readers. We tested the impact of multiple sources of variance on the JAMRIS-TMJ score, estimating not only the impact of differences in readers, but also the differences in levels of reader calibration (i.e., imaging atlas [19], with or without tutorial), reader specialty, and patient-level correlation. Specifically, our primary aim was to compare the scoring system’s inter-reader reliability by subgroups, at different levels of calibration and by radiologist and non-radiologist clinician groups. Secondary aims included the assessment of reliability in less controlled scenarios, where multiple sources of variability coexist in the scoring method. These sources included within-reader variations, between readers with different levels of calibration, between radiologist and non-radiologist clinician groups, as well as the score correlation between the right and left TMJ within the same patient. The analysis was based on generalizability (G) theory [20,21], as it is able to produce comparable reliability coefficients that can integrate these additional sources of variance (for more background information, please see Appendix A). By comparing the reliability coefficients and the relative impact of these variances on the overall measurement error, we proposed recommendations on the reading conditions to improve reliability. 

## 2. Materials and Methods

### 2.1. Scoring Materials and TMJ MRI Exams

The scoring system tested in this study is developed to evaluate the MRI-observable changes in the TMJs in children with JIA (named JAMRIS-TMJ) [16]. It consists of 8 weighted items grouped into inflammatory and osteochondral damage domains. The items are graded in two or three levels, and include bone marrow edema, bone marrow enhancement, joint effusion, synovial thickening, and joint enhancement in the inflammatory domain, and condylar flattening, erosion, and disk abnormalities in the damage domain. The definitions of items and grades of the JAMRIS-TMJ appear in Appendix B. The two TMJs visualized in the same MRI exam are scored independently of the contralateral side.

The TMJ MRI exams used for the reading were performed on a 1.5 Tesla system with dual ring coils in 25 patients and larger multichannel surface coil in 6 patients. The imaging sequences contained T1-weighted, proton density-weighted, and fat suppressed T2-weighted precontrast sequences in the sagittal oblique plane, and T1-weighted fat suppressed Gadolinium-enhanced sequences in the sagittal oblique and coronal planes (Appendix C). A TMJ MRI atlas for JIA that supplements the JAMRIS-TMJ was used in some of the reading groups to study the difference in reader calibration. The atlas included ideal representations and descriptions of each of the scored items and grades in relevant imaging sequences, as well as key image interpretation pitfalls [19]. The reading order of the exams was randomized for each reader and scoring scenario.

Sample size was estimated using reference tables based on the intraclass correlation coefficient (ICC). Assuming 5 readers, using 32 cases would achieve a 95% confidence interval width of 0.2 around an expected ICC of 0.8, or 0.3 around 0.6 [22,23]. In total, bilateral joints from 31 patients were used, with the 62 total joints analyzed in a hierarchically nested model. Scans were chosen nonrandomly from previously imaged patients with known or suspected JIA to represent the full range of TMJ pathology in this condition, from normal appearances to severe inflammation and deformity.

### 2.2. Reading Exercise and Data Structure

**Figure 1:**
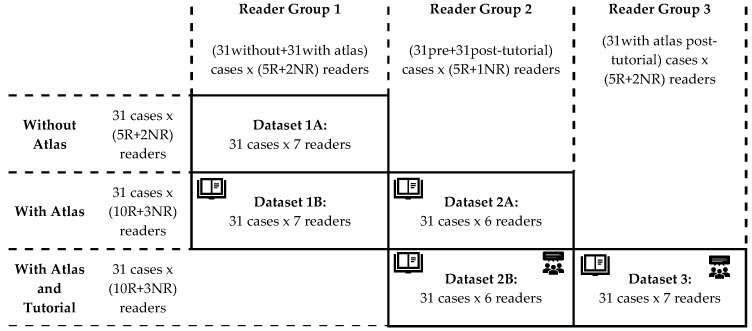
Schematic representation of the composition and methods of the different reader groups. All reader groups in this study used the same set of 31 bilateral temporomandibular joint (TMJ) examinations from patients with diagnosed or suspected juvenile idiopathic arthritis (JIA, clinical characteristics shown on Table 1). Pooling datasets 1B with 2A, and 2B with 3 enables the calculation of inter-reader reliability in larger reader groups and within and between reader specialty groups (results shown on Table 2). Pooling datasets 1A with 1B, and 2A with 2B enables the calculation of intrareader reliability between levels of calibration and inter-reader reliability between and within levels of calibration (results shown on Table 3). Abbreviations: R, radiologist; NR, non-radiologist clinician.

A schematic summary of the study design is shown in Figure 1. Bilateral TMJ MR studies from 31 patients were read by a total of 20 readers (15 radiologists, 2 surgeons, 2 rheumatologists and 1 orthodontist) in three groups blinded to clinical information:

- Group 1 included five radiologists, one oral-maxillofacial surgeon, and one rheumatologist. These seven readers first scored the 31 cases with just the provided scoring system (dataset 1A), then scored the same cases again after 1–2 months, using the imaging atlas (dataset 1B).
- Group 2 included five radiologists and one oral-maxillofacial surgeon. These six readers first scored the same 31 cases using the scoring system and the atlas (dataset 2A), then scored the same cases again after 1–2 months, following a group calibration tutorial session (dataset 2B).
- Group 3 consisted of seven readers including 5 pediatric radiologists, 1 pediatric rheumatologist, and 1 orthodontist who also scored the same cases, but only once, after the group calibration session that was held together with the group 2 readers (dataset 3).

The 13 readers in groups 1 and 2 were randomly assigned to their respective groups. The group 3 readers were analyzed separately, since they had previously participated in a reliability exercise using 21 of these 31 cases and three existing TMJ MRI scoring systems from which this new scoring system was developed [16].

### 2.3. Data Analysis

Reliability of score on a single joint was assessed using G coefficients, which are extensions of the intraclass correlation coefficient (Appendix A). Two- or three-facet G coefficients were calculated based on whether a third variable level for each observation was stratified or pooled, respectively (Figure 1), as described below.

The two-facet G coefficients contain the “Reader” and “Patient” facets and are presented by stratifying the “Aid” variable in three groups (i.e., baseline, atlas, atlas + tutorial levels), and also the “Specialty” variable in three groups (radiologists, non-radiologist clinicians, and total), yielding 3 × 3 matrix of two-facet G coefficients for each domain.

For the three-facet G coefficients, in addition to the “Rater” and “Patient” facets, a third facet, either the “Aid” or “Specialty”, is included in the calculation. The five datasets are pooled according to the third facet variable by:

1. Combining the different calibration levels while keeping radiologist and non-radiologist groups separate, i.e., dataset 1A with 1B for +/−atlas, and 2A and 2B for +/−tutorial (vertical pooling on Figure 1).
2. Combining the radiologist and non-radiologist data while keeping the calibration level separate, i.e., dataset 1B with 2A, and 2B with 3 (horizontal pooling on Figure 1).

### 2.4. Statistical Methods

Multiway ANOVA was performed using the VARCOMP procedure with the restricted maximum likelihood method in SAS 9.4 (Cary, NC, USA) to determine the variance components corresponding to the main effect and interactions of the clustering variables in this study, which are the joint (J), patient (P), reader (R), the presence or absence of aid (A), and whether the reader was a radiologist or non-radiologist clinician (S, for specialty). Using these variance components, G coefficients corresponding to various types of measurement scenarios were calculated by the formulae in Appendix D, which were derived from references on G theory analysis [20,21].

## 3. Results

The clinical characteristics of the patient sample are listed in Table 1. There was a high prevalence of females (84%) and the oligoarticular subtype of JIA (55%). On MRI, by median of 13 tutorial-calibrated readers, 71% of joints showed nonzero grade for the JAMRIS-TMJ inflammation domain (range 55–95%, IQR 68–79%), and 69% for the damage domain (range 47–81%, IQR 58–74%). Unilateral inflammatory findings, i.e., non-zero inflammation domain score only on one side, was seen in 19% of patients (range 10–32%, IQR 13–23%); unilateral osteochondral damage was also seen in 19% of patients (range 6–35%, IQR 13–26%).

### 3.1. Two-Facet G Coefficients: Reliability by Subgroups of Measurement Aid and Reader Specialty

Table 2 lists the results of reliability in each of the reader subgroups. The inter-reader reliability coefficients in the typical research setting, where multiple radiologists score the images with the aid of the atlas and after an interactive calibration tutorial, were 0.73 for the inflammatory and 0.77 for the damage domain (Table 2). These correspond to a 95% measurement error of +/− 25 percentage points each on the respective JAMRIS-TMJ domains.

When the radiologist and non-radiologist clinician groups were pooled together, the atlas (13 readers) and the atlas + tutorial (13 readers) cohorts showed increasing inter-reader reliability compared to the baseline cohort (7 readers). For the radiologist subgroups, the inter-reader reliability did not change with the use of the atlas and with the addition of a tutorial for both the inflammatory and damage domains. For the non-radiologist clinician subgroups, the inter-reader reliability for both domains were lower than those of radiologists.

### 3.2. Multiway ANOVA: Contextual Impact of Calibration Level and Reader Specialty

The variance components obtained through multiway ANOVA (Appendix E) showed that the score given to a TMJ was modified non-trivially by the reader rating the images, their specialty group, as well as the level of reading aid used (calibration level). The main aid-related variance component by itself was small and insignificant. However, the three-way interaction terms involving the aid, reader, and patient variables showed statistical significance (*p* < 0.0001) for the radiologist readers, suggesting that the atlas and tutorial caused context-specific changes to the TMJ score in some reader-patient combinations. The equivalent interaction effect in the non-radiologist clinical group did not reach statistical significance after Bonferroni correction, despite showing higher variance components to the radiologists’ data (18 vs. 13% of the total variance for the inflammation score, and 7 vs. 6% for the damage score), likely owing to the lower number of non-radiologist participants. Furthermore, for the damage domain, the aid*reader interaction was significant, suggesting that some readers rated all cases higher in general after the tutorial.

When pooling across different reader specialty groups (i.e., *n* = 7, 13, or 13 readers across the three calibration levels), there was a significant systematic difference associated with the readers for both domains and all calibration levels, meaning some readers systematically gave higher grades across all patients. However, this was not correlated with whether the reader was a radiologist or non-radiologist, since the specialty main effect and patient*specialty interaction were not significant. The interaction terms with reader and patient were significant, suggesting that some readers scored the two joints of the same patient more similarly than other readers.

### 3.3. Three-Facet G coefficients: Reliability When Variations in Calibration or Specialties Exist in the Dataset

Table 3 describes the reliability when some measurement characteristics are not controlled, such as when not all the readers have attained the same calibration level or that readers from different specialties are participating in the reader cohort. Agreement between radiologists belonging to the same calibration level ranged from 0.69–0.81 for the two domains and two calibration gradients (Table 3, data row 1). The opposite scenario, which is the agreement within the same reader between the use and disuse of a calibration aid, was higher, ranging from 0.77–0.88 for the two domains and two calibration gradients (row 2). The combination of these sources of error, i.e., when comparing different readers who also differ in their level of calibration, the agreement ranged from 0.68–0.78 (row 3). When keeping the calibration level variable constant to estimate the impact of reader specialty, the agreement between radiologists and non-radiologist clinicians ranged between 0.56–0.70 (row 9). Agreement among readers of the same specialty was higher, ranging between 0.67–0.76 (row 10). In terms of measurement error, the presence of heterogeneity in the level of calibration and reader specialty widen the measurement error by up to 4% and 7%, respectively (row 1 vs. 3, and 9 vs. 10).

Agreement on the domain score between the right and left joint of the same patient was generally low but not absent. In the most reliable measurement scenario, i.e., when assessed by the same reader within the same level of calibration (rating both joints in the same sitting), the right−left correlation ranged from 0.30–0.49, or approximately within 45–51% domain score points in 95% of cases (Table 3 rows 8 and 12). In the least reliable scenario, i.e., when both the reader and the level of calibration or specialty differed, the agreement still ranged between 0.12–0.39 (contralateral TMJ score within 53–65% points in 95% of cases, rows 7 and 11). In general, the right−left joint correlation was higher for the damage domain.

### 3.4. Variation of Reliability by Study Design Differences

For assessing the impact of sources of variabilities in different study designs, as well as to calculate the sample size needed to detect a hypothesized level of difference, the potential level of measurement error of JAMRIS-TMJ can be estimated using the G coefficient formulas in Appendix D and variance component estimates specific to the model, such as in Appendix E. Between-reader variance was a much greater source of measurement variability when compared to within-reader changes due to calibration aid. The latter consists of both the random intrareader variations over time in addition to any systematic change in score caused by the atlas or the tutorial. In study designs where measurements are taken in replicate to reduce measurement error, it will thus be more beneficial to average scores across different readers, rather than averaging multiple scores given by the same reader (provided at different states of calibration). For example, considering the use case where the reliability of readers with different levels of calibration is 0.78 for the damage domain (95% CI of measurement at +/− 25 percentage points), averaging two different readers achieves 0.88 (+/−19%), whereas averaging two readings of the same reader achieves 0.84 (+/−20%).

## 4. Discussion

Our study assessed the reliability of a tool for the semiquantitative grading of TMJ arthritis, JAMRIS-TMJ, as well as the relative impact of various potential sources of measurement error in its application. In the most controlled and typical use case, i.e., a group of radiologists grading with the atlas and after a calibration tutorial, the true score is expected to be within +/−25 percentage points of any given score 95% of the time (Table 2). The atlas and tutorial caused significant contextual changes in the reader’s assessment of the joints as per the ANOVA results. However, the variable presence of this effect did not further improve the group-level clustering of scores among radiologists. Instead, the impact of calibration aids was limited to improving the agreement between radiologist and non-radiologist clinician readers. It is important to note that calibration is nevertheless required to improve the accuracy of scores irrespective of its effect on improving reliability, since the two are independent characteristics of measurement error. 

**Figure 2:**
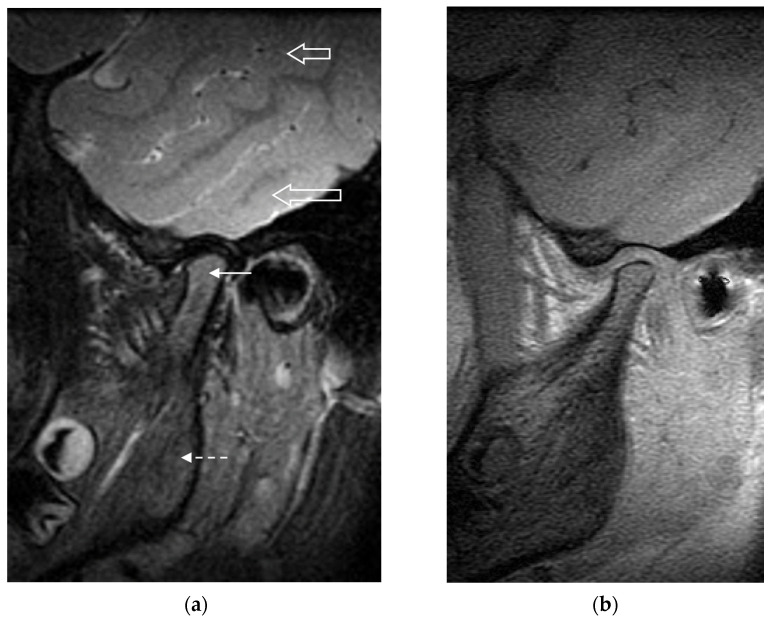
(**a**) Sagittal T2-weighted fat suppressed MR image of the left temporomandibular joint in a 9-year-old girl. The signal intensity of the bone marrow in the mandibular condyle (solid arrow) is increased compared to the signal intensity of that in the mandibular ramus (dashed arrow). Following the exact definition of bone marrow edema in the scoring system would cause this to be scored as grade 1 (present) bone marrow edema. However, this increased signal intensity is likely secondary to the generally higher signal in structures at the center of the field of view versus those at the periphery: notice the higher signal intensity of brain parenchyma nearer the center of the image (lower open arrow) versus that at the periphery of the image (upper open arrow). If this were true marrow edema, the signal intensity on the corresponding precontrast T1-weighted image (**b**) would be expected to be decreased, which was not the case.

The reliability results observed in this study are comparable to the moderate-to-good range of results seen with other TMJ MRI scoring systems published in the literature [16,24,25]. Compared to larger joints such as the knees and hips, grading change in the TMJ on MRI may be less reliable due to the limitations in image resolution and the TMJ’s anatomical complexity. The small size of the TMJ reduces the score range in which the TMJ can be graded, by limiting both the number of definable disease features as well as the range of their grading. This quantitative limitation in turn reduces the between-patient variance relative to other variances in measurement, leading to reduced measurement reliability coefficients. Furthermore, despite best efforts to specify the definitions and representation in the JAMRIS-TMJ, it remains challenging to identify, differentiate, and grade the features. Some specific issues that introduce subjectivity in scoring the inflammatory changes include the physiological age-related conversion of hematopoietic bone marrow, the nonuniformity of signal across the surface coil (Figure 2), and differentiating the inflamed synovium from the joint fluid. The structural changes also remain challenging to score, as the patient-referenced normal joint shape is often unavailable and would need to be assumed and imagined by the reader to serve as reference for grading flattening and erosions.

A further step in investigating the reliability of JAMRIS-TMJ is to also quantify the patient- and imaging-related changes over time. Changes in the TMJ score between repeat imaging of the same state of disease may be significant when the imaging parameters are not standardized, or the imaging interval is long enough to introduce physiological changes. Quantitative methods for scoring the degree of inflammation have demonstrated a high degree of discriminatory validity [26,27] but are also affected by temporal variations [28]. In a semiquantitative scoring system such as the JAMRIS-TMJ, these errors may be relatively low compared to the between-reader variance but should still be accounted for when using the scoring system in longitudinal evaluation. Another type of variance that may be important to identify is the impact of comparing to the contralateral TMJ on the score. The patient variance component in this study does not differentiate how much patient-wise correlation is due to the pathophysiological factors that cause the two sides to be correlated, and how much is due to the reader intentionally adjusting the joint score by comparing to the contralateral side. A more sophisticated study design utilizing artificially paired right and left TMJ exams would be able to identify the magnitude of this effect, which may be helpful for improving the grading of items that require a within-patient comparator.

Our study conclusions should be interpreted in the context of several potential limitations. One limitation is that methods for calculating confidence intervals for these more complex types of G coefficients are not yet available and currently limited to the simplest one-facet crossed design [21]. The point estimates of various G coefficients presented in this paper should be used for identifying trends in the relative impact of quality controls in the measurement and on the estimation of sample size. Secondly, although the group 1 and 2 readers read the same cases twice, there was a change in the aid they used, which makes the coefficient a “within-reader, between-aid” agreement, rather than the traditional intrareader reliability where there are no external changes to the measurement scenario. However, it is reasonable to assume that the intrareader reliability will be at least as high as the within-reader, between-aid reliability since the latter is additionally lowered by any systematic variations attributable to the use and disuse of the aid.

## 5. Conclusions

In summary, this study demonstrates that semiquantitative MRI scoring of TMJ arthritis using the JAMRIS-TMJ is reliable in the calibrated setting, particularly when performed as a double-read by two radiologists, forming the foundation for its potential use in the clinically important assessment of change over time and with therapy. The use of atlas and tutorial calibration is recommended when multiple specialty groups are participating in reading.

## Figures and Tables

**Table 1:** Clinical characteristics of the cohort of 31 patients whose MRI examinations of temporomandibular joints (TMJs) were used for the reliability exercise. Laboratory and physical examination test values are those available at the closest date within three months in relation to the study MRI date. Abbreviations: JIA (juvenile idiopathic arthritis), SD (standard deviation), HLA-B27 (human leukocyte antigen B27), ANA (antinuclear antibody), RF (rheumatoid factor), DMARD (disease modifying antirheumatic drug), anti-TNF (tumor necrosis factor inhibitor).

Clinical Characteristics of Patient Sample	
Age at diagnosis (years)	8.0 (SD 4.5, range 0.5–15.3)
Age at MRI (years)	11.6 (SD 3.0, range 6.2–16.9)
Disease duration (years)	3.6 (SD 4.4, range 6.8–15.7)
Sex	5 male, 26 female
JIA subtype	
Oligoarticular	12
Oligoarticular extended	5
Polyarticular	9 (all RF−)
Enthesitis related	1
Psoriatic arthritis	1
Undifferentiated	1
No JIA diagnosis	2
HLA-B27+ (*n* tested, % of tested)	1 (19, 5%)
ANA+	18 (58%)
RF+ (*n* tested, % of tested)	1 (30, 3%)
Uveitis	7 (23%)
Facial changes (including asymmetry, decreased condylar translation, retrognathia)	19 (61%)
Crepitation	4 (13%)
Decreased mouth opening (<10th percentile)	10 (32%)
TMJ pain	7 (23%)
Active treatment (including NSAIDs, DMARDs, anti-TNF, etc.)	20 (65%)
History of DMARD use (past and/or current)	15 (48%)

**Table 2:** Two-facet generalizability coefficients. Agreement on the TMJ score with two sources of variance—patient and reader. The 95% confidence interval of measurement reflects the measurement error interval around a given score, in the units of the respective JAMRIS domain.

Inter-Reader Absolute Agreement Reliability	Generalizability Coefficient			95% CI of Score (+/− Percentage Points)		
	Baseline	Atlas	Atlas + Tutorial	Baseline	Atlas	Atlas + Tutorial
**Inflammatory domain**						
Radiologists (*n* = 5, 10, 10)	0.71	0.73	0.73	27	26	25
Non-radiologist clinicians (*n* = 2, 3, 3)	0.49	0.53	0.45	35	30	37
All readers (*n* = 7, 13, 13)	0.61	0.66	0.67	32	28	28
**Damage domain**						
Radiologists (*n* = 5, 10, 10)	0.76	0.77	0.77	24	25	25
Non-radiologist clinicians (*n* = 2, 3, 3)	0.44	0.46	0.70	42	42	28
All readers (*n* = 7, 13, 13)	0.68	0.66	0.74	29	31	27

**Table 3:** Three-facet generalizability coefficients. Agreement on TMJ score with three sources of variance—patient, reader and aid or reader specialty. The 95% confidence interval of measurement reflects the measurement error interval around a measured score, in the units of the respective JAMRIS-TMJ domain. Abbreviations: A, aid; J, temporomandibular joint; Non-Rad, non-radiologist clinician reader; P, patient; R, reader; Rad, radiologist reader; S, specialty (binary, radiologist or non-radiologist clinician).

Pooling the Two Readings from the Same Readers	Generalizability Coefficient						95% CI of Measurement (+/− Percentage Points)					
	Inflammatory Domain			Damage Domain			Inflammatory Domain			Damage Domain		
Measurement Scenario (Main Effects Definitions)	+/− Atlas		Atlas +/− Tutorial Rad	+/− Atlas		Atlas +/− Tutorial Rad	+/− Atlas		Atlas +/− Tutorial Rad	+/− Atlas		Atlas +/− Tutorial Rad
	Non-Rad	Rad		Non-Rad	Rad		Non-Rad	Rad		Non-Rad	Rad	
Between readers of the same calibration (R random, P and A fixed)	0.49	0.69	0.81	0.39	0.78	0.80	35	28	21	47	24	23
Same reader with vs. without aid (A random, P and R fixed)	0.65	0.86	0.83	0.59	0.77	0.88	29	17	20	33	24	18
Between readers with different calibration (A and R random, P fixed)	0.41	0.67	0.75	0.37	0.76	0.78	37	29	25	48	25	25
Between readers with different calibration, averaging two readings per reader (A and R random, P fixed, A/2)	0.50	0.73	0.83	0.43	0.86	0.84	31	26	20	42	18	20
Between readers with different calibration, averaging two readers at a time (A and R random, P fixed, R/2)	0.56	0.81	0.84	0.53	0.86	0.88	28	21	19	35	18	17
Between readers of the same calibration, averaging two readers at a time (R random, P and A fixed, R/2)	0.65	0.82	0.90	0.56	0.87	0.89	25	20	15	34	17	16
Between right and left joints when scored by different readers with different calibration (J, A and R random)	0.12	0.20	0.27	0.23	0.39	0.34	54	56	55	65	54	56
Between right and left joints when scored by the same reader with the same calibration (J random, A and R fixed)	0.30	0.39	0.43	0.39	0.47	0.44	48	47	47	51	50	50
**Pooling the Readings from Same Calibration**	**Generalizability Coefficient**						**95% CI of Measurement**					
**Measurement Scenario (Main Effects Definitions)**	**Inflammatory Domain**			**Damage Domain**			**Inflammatory Domain**			**Damage Domain**		
	**Baseline**	**Atlas**	**Tutorial**	**Baseline**	**Baseline**	**Atlas**	**Tutorial**	**Baseline**	**Baseline**	**Atlas**	**Tutorial**	**Baseline**
Between radiologists and non-radiologists (R and S random, P fixed)	0.56	0.59	0.63	0.64	0.61	0.70	36	31	29	30	33	28
Between readers of the same specialty (R random, S and P fixed)	0.67	0.69	0.67	0.68	0.70	0.76	29	27	28	29	29	26
Between right and left joints when scored by a radiologist and a clinician (J, R and S random)	0.12	0.25	0.26	0.35	0.32	0.30	60	53	53	56	55	56
Between right and left joints when scored by the same reader (J random, R and S fixed)	0.34	0.43	0.40	0.49	0.41	0.42	48	45	46	49	50	50

## Data Availability

Data presented in this study are available on request from the corresponding author.

## Appendix A. Background Information on Generalizability Theory as Applied to Imaging

Reliability studies have often used the intraclass correlation coefficient (ICC) deriving from classical test theory, which models measurements by two components—the true score and error. This approach is limited to the analysis of a single source of measurement error. In the typical inter-reader reliability study where multiple readers score the same set of images, one- or two-way analysis of variance (ANOVA) is used to determine how much of the variance in the study data is associated with the image variable (the true score component), and how much is associated with the reader variable (part of the error component). However, there are often more than one source of variance that may be important to analyze in an instrument’s typical use case. For example, assessment of systemic treatments in arthritis may require the scoring of multiple joints from each patient, especially for the bilateral joints, such as the TMJs. The existence of any patient-level correlation in the score variance introduces a source of clustering in the data which must be analyzed as a fixed effect under the assumptions regarding the independence of data for common statistical tests. Other sources of variance may exist in the study sample, including differences in the imaging protocols and equipment used to acquire the exams, training background and experience of the readers, as well as the measurement aids or calibration tutorials used for the scoring. If using the traditional ICC, the study sample would need to be stratified at each level of these variations to study these other sources of variance, or assume such variances do not exist.

A more comprehensive approach called the generalizability theory allows the estimation of an overall ICC that is generalized over multiple sources of variability [20,21]. The generalizability study (G-Study) extends upon the CTT definition of ICC by using multiway ANOVA, which allows the quantification of more than two sources of variance (called facets) and their interactions. When these are known, it is possible to calculate additional reliability coefficients that may be important in order to understand the measurement variability across common variations in measurement methods. The design of such a G-Study will require measuring the same data under different conditions and pooling the dataset across the levels of the generalizing variables. For example, in an inter-reader study where each reader also read the same MRI exam twice, a G-study can calculate both the inter-reader and intrareader reliability coefficient using the complete set of data, without the need to create different subgroups for the two coefficients, hence maintaining study power and improving external validity. Furthermore, the results of the multiway ANOVA identify the relative impact of the sources of variance to the overall measurement error, which allows for the optimization of study designs by simulating the measurement conditions (at an analysis step called the design study, or D-Study). For example, it will be possible to compare the expected reliability between averaging different readers’ score for each joint versus averaging multiple readings done by the same readers, allowing the researchers to choose a design that best minimizes the number of measurements needed to meet a priori study power and effect size thresholds.

## Appendix B. JAMRIS-TMJ Scoring System

**Table A1:** The scoring system tested in the exercise is referred to as the Juvenile Idiopathic Arthritis Magnetic Resonance Scoring System for Temporomandibular Joints (JAMRIS-TMJ). The semiquantitative grades are weighted per domain to yield domain scores which are scaled from 0–100% [17]. The two temporomandibular joints are graded separately.

**INFLAMMATORY DOMAIN**					
**Definition**	**Bone Marrow Edema**	**Bone Marrow** **Enhancement**	**Effusion**	**Synovial Thickening**	**Joint Enhancement**
	Compared to the mandibular ramus, hyperintense marrow signaling within the condyle on T2w FS or STIR images, and/or hypointense signaling on pre-contrast T1w images without FS.	Compared to the mandibular ramus, hyperintense marrow signaling within the condyle on post-contrast T1w FS images.	Increased joint fluid with isointense signaling of joint space compared to that of cerebrospinal fluid on T2w FS or STIR images.	Thickened synovial lining of the TMJ with intermediate signal on T2w images.	Signal intensity of the synovium, capsule, and joint fluid higher than that of muscle on post-contrast T1w FS images.
**Grading**	Absent	Absent	Normal: ≤1mm fluid in joint recess	Absent: No synovium visible (joint space ≤1 mm width)	Normal: High signal intensity confined to signal perimeter of normal amount of joint fluid on corresponding fluid-sensitive image
	Present	Present	Small: >1 and ≤2mm fluid in recess or involving entire joint compartment	Mild: >1 and ≤2mm thickness at the point of maximum synovial thickening	Mild: High signal intensity focally exceeding signal perimeter of physiologic amount of joint fluid on corresponding fluid-sensitive image
			Large: >2mm fluid in recess or involving entire joint compartment	Moderate/Severe: >2mm thickness at the point of maximum synovial thickening	Moderate/Severe: High signal intensity diffusely involving one or both joint compartments
**DAMAGE DOMAIN**					
**Definition**	**Condylar Flattening**		**Erosions**		**Disk Abnormalities**
	Loss of the round or slightly rectangular shape of the condylar head, viewed in the sagittal-oblique plane.		Any irregularity or breaks of the bony joint surfaces leading to the loss of the smooth continuous surface of the bone, seen in both sagittal and coronal planes		Any abnormality of the articular disk, including flattening, displacement, or destruction.
**Grading**	Normal round/slightly rectangular shape		No irregularities or deep breaks		Absent
	Mild: Extent of flattening involves part of the surface of the condyle		Mild: Presence of irregularities involving only part of the articular surface of the condyle		Present
	Moderate/Severe: Extent of flattening involves the entire surface of the condyle, or loss of height in the condyle.		Moderate/Severe: Presence of deep breaks in the subchondral bone seen in two planes, or irregularities involving the entire articular surface of the condyle		

## Appendix C. Imaging Protocol

**Table A2:** Representative MRI protocol for the TMJ exams used in the reliability exercise. Abbreviations: FOV, field-of-view; FS, fat suppression sequence; FSE, fast spin echo; FSPGR, fast spoiled gradient recalled echo; + Gd, post gadolinium injection; mm, millimeter; ms, milliseconds; PD, proton density-weighted sequence; SE, spin echo.

	Imaging Sequence (in Order of Acquisition from Left to Right)					
	T1 FSPGR	PD FSE	T2 FSE FS	T1 FSE FS + Gd	T1 SE FS + Gd	3D FSPGR + Gd
Plane	Sagittal oblique	Sagittal oblique	Sagittal oblique	Sagittal oblique	Coronal	Sagittal oblique
Echo time (ms)	4.2	25	86	11	19	10.4
Repetition time (ms)	325	2660	2840	600	600	4.2
Flip angle	80	90	90	90	90	20
FOV (mm × mm)	120	120	120	120	160	100
Acquisition Matrix	384 × 224	256 × 224	256 × 224	256 × 224	256 × 192	256 × 192
Slice thickness (mm)	2	2	2	2	2	2
Slice spacing (mm)	2	2	2	2	2	1
Echo train length	-	8	16	3	-	-

## Appendix D. Generalizability Coefficient Formulae

**Table A3:** Formulae used to calculate the G and Φ generalizability coefficients in Table 3, derived from references [20,21]. Abbreviations: A, aid or calibration level; J, temporomandibular joint; P, patient; R, reader; S, specialty (binary, radiologist or non-radiologist clinician); colon (:), nested relation, (e.g., J:P means joint is nested within the patient variable); cross (x), crossed relation (e.g., RxA means each Reader provides data at all levels of the aid variable).

**Coefficient Meaning**	Formula
J:P × R × A Design—Pooling Across Use or Disuse of Aid, Separately for Radiologist and Non-Radiologist Readers	
Between readers of the same calibration (R random, P and A fixed)	$G\left(R\right)=\frac{J:P+AJ:P}{J:P+AJ:P+R+RJ:P+ARJ:P}$
Same reader with vs. without aid (A random, P and R fixed)	$G\left(A\right)=\frac{J:P+RJ:P}{J:P+RJ:P+A+AJ:P+ARJ:P}$
Between readers with different calibration (A and R random, P fixed)	$G\left(R,A\right)=\frac{J:P}{J:P+A+R+AR+AJ:P+RJ:P+ARJ:P}$
Between readers with different calibration, averaging 2 readings per reader (A and R random, P fixed, A/2)	$G\left(R,A\right)=\frac{J:P}{J:P+\frac{A}{2}+R+\frac{AR}{2}+\frac{AJ:P}{2}+RJ:P+\frac{ARJ:P}{2}}$
Between readers with different calibration, averaging 2 readers at a time (A and R random, P fixed, R/2)	$G\left(R,A\right)=\frac{J:P}{J:P+A+\frac{R}{2}+\frac{AR}{2}+AJ:P+\frac{RJ:P}{2}+\frac{ARJ:P}{2}}$
Between readers of the same calibration, averaging two readers at a time (R random, P and A fixed, R/2)	$G\left(R\right)=\frac{J:P+AJ:P}{J:P+AJ:P+\frac{R}{2}+\frac{RJ:P}{2}+\frac{ARJ:P}{2}}$
Right-left joint agreement when scored by different readers with different calibration (J, A and R random)	$\Phi \left(J,R,A\right)=\frac{P}{P+J:P+R+A+PA+PR+AR+PAR+AJ:P+RJ:P+ARJ:P}$
Right-left joint agreement when scored by the same reader with the same calibration (J random, A and R fixed)	$G\left(J\right)=\frac{P+PA+PR+PAR}{P+PA+PR+PAR+J:P+AJ:P+RJ:P+ARJ:P}$
**J:P × R:S Design—Pooling Across Radiologist and Non-Radiologist Readers, Separately for Each Aid**	
Between radiologists and non-radiologists (R and S random, P fixed)	$G\left(R,S\right)=\frac{J:P}{J:P+R:S+S+J:PS+JR:PS}$
Between readers of the same specialty (R random, S and P fixed)	$G\left(R\right)=\frac{J:P+J:PS}{J:P+J:PS+R:S+JR:PS}$
Right-left joint agreement when scored by radiologist and non-radiologist (J, S and R random)	$\Phi \left(J,R,S\right)=\frac{P}{P+J:P+R:S+S+PS+PR:S+J:PS+JR:PS}$
Right-left joint agreement when scored by the same reader (J random, R and S fixed)	$G\left(J\right)=\frac{P+PS+PR:S}{P+PS+PR:S+J:P+J:PS+JR:PS}$

## Appendix E. Multiway ANOVA Results

**Table A4:** Analysis of the JAMRIS-TMJ scores by multiway ANOVA, where all effects are modeled as random. In the J:P × R × A design (top half), the effects of three factors, patient, aid, and reader on the joint score are analyzed by combining the 1A and 1B datasets under the atlas column, and the 2A and 2B datasets under the tutorial column from the radiologists. In the J:P × R:S design, the effect of patient, reader and specialty are analyzed by combining the 1B and 2A datasets for the atlas row, and 2B and 3 datasets for the tutorial row, with 1A as baseline. Bolded *p* values are those which remained significant after applying Bonferroni correction for multiple testing.

J:P × R × A Design	Inflammatory Domain						Damage Domain					
	Atlas Radiologists		Atlas Non-Radiologists		Tutorial Radiologists		Atlas Radiologists		Atlas Non-Radiologists		Tutorial Radiologists	
Variance Component	Var Comp	*p*	Var Comp	*p*	Var Comp	*p*	Var Comp	*p*	Var Comp	*p*	Var Comp	*p*
Joint (patient)	44%	**<0.0001**	27%	0.00	46%	**<0.0001**	40%	**<0.0001**	24%	0.00	46%	**<0.0001**
Patient	20%	0.05	14%	0.11	27%	0.02	38%	0.00	23%	0.01	34%	0.01
Reader	8%	0.03	0%	0.30	3%	0.06	0%	0.91	19%	0.00	4%	0.05
Aid	0%	0.41	2%	0.40	3%	0.04	1%	0.19	1%	.	0%	0.44
Reader*joint (patient)	4%	**<0.0001**	16%	0.00	1%	0.16	0%	0.66	4%	0.14	0%	0.32
Aid*joint (patient)	0%	0.75	5%	0.09	0%	0.34	0%	0.86	2%	0.29	0%	0.93
Reader*Patient	2%	0.13	1%	0.45	5%	0.00	0%	0.43	3%	0.25	3%	0.00
Aid*patient	0%	0.63	0%	0.91	0%	0.34	1%	0.19	0%	0.74	0%	0.33
Aid*reader	2%	0.00	0%	0.34	1%	0.00	1%	0.00	0%	0.98	2%	**<0.0001**
Aid*reader*patient	13%	**<0.0001**	18%	0.00	8%	**<0.0001**	6%	**<0.0001**	7%	0.06	4%	**<0.0001**
Residual	8%	.	17%	.	7%	.	12%	.	18%	.	7%	.
**J:P × R:S Design**	**Baseline**		**Atlas**		**Atlas+Tutorial**		**Baseline**		**Atlas**		**Atlas+Tutorial**	
**Variance Component**	**Var Comp**	***p***	**Var Comp**	***p***	**Var Comp**	***p***	**Var Comp**	***p***	**Var Comp**	***p***	**Var Comp**	***p***
Joint (patient)	38%	**<0.0001**	35%	**<0.0001**	38%	**<0.0001**	34%	**<0.0001**	36%	**<0.0001**	43%	**<0.0001**
Patient	11%	0.13	24%	0.01	27%	0.01	35%	0.00	32%	0.01	29%	0.01
Reader (specialty)	5%	**<0.0001**	7%	**<0.0001**	6%	**<0.0001**	5%	**<0.0001**	5%	**<0.0001**	4%	**<0.0001**
Specialty	9%	0.07	1%	0.21	0%	0.84	0%	0.79	0%	0.33	1%	0.19
Patient*specialty	0%	0.88	0%	0.53	1%	0.31	3%	0.08	0%	0.84	1%	0.15
Patient*reader (specialty)	19%	**<0.0001**	15%	**<0.0001**	11%	**<0.0001**	9%	**<0.0001**	7%	**<0.0001**	8%	**<0.0001**
Specialty*joint (patient)	2%	0.07	5%	**<0.0001**	3%	0.01	2%	0.05	7%	**<0.0001**	2%	0.00
Residual	16%	.	13%	.	15%	.	12%	.	14%	.	11%	.
